# Early Exposure to High-Sucrose Diet Leads to Deteriorated Ovarian Health

**DOI:** 10.3389/fendo.2021.656831

**Published:** 2021-04-19

**Authors:** Giuliane Barros de Melo, Jéssica Furtado Soares, Thamyres Cristhina Lima Costa, Renata Ohana Alves Benevides, Caroline Castro Vale, Antonio Marcus de Andrade Paes, Renato Simões Gaspar

**Affiliations:** ^1^ Laboratory of Experimental Physiology, Department of Physiological Sciences, Biological and Health Sciences Centre, Federal University of Maranhão, São Luís, Brazil; ^2^ Health Sciences Graduate Program, Biological and Health Sciences Centre, Federal University of Maranhão, São Luís, Brazil; ^3^ Institute for Cardiovascular and Metabolic Research, School of Biological Sciences, University of Reading, Reading, United Kingdom

**Keywords:** metabolic syndrome, diet rich in sucrose, hyperglycaemia, ovarian dysfunction, polycystic ovary syndrome

## Abstract

**Background:**

The metabolic syndrome (MetS) is correlated with disorders of the reproductive system, such as the polycystic ovary syndrome (PCOS). While consumption of a diet rich in carbohydrates is linked to the development of MetS, it is still unclear if this diet leads to ovarian dysfunction and PCOS.

**Objectives:**

We investigated the influence of a high-sucrose diet (HSD) on the ovarian milieu of Wistar rats and studied the correlation between high consumption of sugary drinks and the prevalence of PCOS in women.

**Methods:**

Wistar rats were given a standard laboratory diet (CTR, 10% sucrose, n = 8) or HSD (HSD, 25% sucrose, n = 8) from postnatal day 21 to 120. Animals were evaluated weekly to calculate food intake, feed efficiency and weight gain. Both onset of puberty and estrous cycle were monitored. Metabolic serum biochemistry, organ morphometry and ovarian histology were performed upon euthanasia. In parallel, a fixed-effects multiple linear regression analysis was performed using data from Brazilian states (459 state-year observations) to test the correlation between the consumption of sugar-sweetened beverages (surrogate for HSD intake) and the prevalence of PCOS (surrogate for ovarian dysfunction).

**Results:**

HSD animals showed increased adipose tissue accumulation, hyperglycaemia and insulin resistance when compared to CTR. Interestingly HSD rats also entered puberty earlier than CTR. Moreover, ovaries from HSD animals had an increased number of atretic antral follicles and cystic follicles, which were correlated with the hypertrophy of periovarian adipocytes. Finally, there was a positive correlation between the intake of sugary drinks and prevalence of PCOS in women of reproductive age.

**Conclusions:**

HSD ingestion leads to ovarian dysfunction in rats and could be correlated with PCOS in women, suggesting these alterations could lead to public health issues. Therefore, we reinforce the deleterious impact of HSD to the ovarian system and suggest that the reduction of added sugars intake could be beneficial to ovarian health.

## Introduction

Metabolic syndrome (MetS) is defined as the presence of at least three of the following: obesity, insulin resistance (IR), dyslipidaemia, hypertension and increased fasting glycaemia (1). MetS prevalence ranges from 10 to 40% worldwide, posing it as an alarming epidemic (2, 3). Of importance, comorbidities associated with MetS have also increased globally in children (4), being linked to higher consumption of sugar-enriched foods, which correspond to nearly 25% of the total energy intake in childhood (5, 6). Indeed, in developing countries, such as Brazil, over 60% of the population was reported to consume excessive amounts of added sugars (7). This same study showed that women consumed more sugar when compared to men. In parallel, it is estimated that 16.8% of men and 24.4% of women are obese in Brazil (8), suggesting that high prevalence of obesity is associated with excessive intake of dietary sugars. This is of particular importance, since MetS and obesity generate a high burden to both the individual and the health care system because of their association with increased risk of developing cardiovascular diseases, non-alcoholic fatty liver disease and reproductive disorders (9).

The link between MetS and reproductive disorders is multifaceted due to hormonal imbalances. For instance, it has been reported that hyperinsulinemia and IR are involved with hyperandrogenism, since insulin stimulates the production of ovarian androgens in synergy with luteinizing hormone (10). Both hyperandrogenism and IR are intrinsically related to ovarian disorders, such as the polycystic ovary syndrome (PCOS), which is characterized by the presence of at least two of the following: irregular menstrual cycles, hyperandrogenism and polycystic ovaries (11). In addition, female obesity is associated with increased concentrations of leptin in serum and follicular fluid and decreased serum levels of adiponectin, which consequently causes or worsens infertility (12). Since the prevalence of obesity among women of reproductive age has been increasing in recent years (13), more efforts are needed to understand the consequences of obesity on ovarian function.

In this regard, the effects of high-fat diet (HFD) consumption have been studied in rodents. HFD ingestion is reported to cause irregular estrous cycle, decreased fertility rates and impaired follicular development, while increasing ovarian inflammation and leptin levels (14). Moreover, it has been proposed that both the quantity and quality of total carbohydrate consumption are also related to infertility in women, reiterating that insulin and glucose metabolism are important factors in female fertility (15). Recently, it has been reported that among women attempting to conceive in Denmark and North America, consumption of diets high in glycaemic load, carbohydrate-to-fiber ratio, and added sugars were negatively associated with reduced fecundability (16). However, there are still very few basic, clinical or epidemiological studies exploring the association of high-sugar dietary intake and reproductive disorders in females.

Therefore, taking into account the high prevalence of obesity amongst women of reproductive age and the scarcity of studies in rodents and humans that explore the effects of high-sucrose diet (HSD) intake to ovarian function, we hypothesized that HSD ingestion leads to ovarian dysfunction. Of note, the HSD used in this study mimics dietary intake of sucrose found in Latin American countries (7). Our main objective was to determine if HSD could lead to ovarian dysfunction. To address this question, we have measured biochemical, morphometric and histological parameters in female rats after exposure to HSD from weaning to 120 days-old. In addition, we sought to correlate the ingestion of HSD with ovarian dysfunction in an ecologic study, to assess the epidemiologic relevance of data found in rodents. Our data shows that HSD intake was deleterious to the ovarian function of rats, while there was a positive correlation between high-sugar intake and prevalence of PCOS in women of reproductive age.

## Materials and Methods

### Animals

Female Wistar rats (Rattus norvegicus) with 21 days of age and approximately 45 g were used, supplied by the Animal Facility House of the Federal University of Maranhão (UFMA) and kept in the Bioresources Unit of the Experimental Physiology Laboratory. Animals were kept at constant temperature (23 ± 2°C), 12h light/dark cycle and *ad libitum* access to water and food. All experimental procedures were performed in accordance with the rules of Brazilian Council for the Control of Animal Experimentation and approved by the Ethical Committee on Animal Use and Welfare at UFMA, ruling number 23115.007440/2016-71.

### Preparation of High-Sucrose Diet

HSD was manufactured with powdered standard chow (40%), condensed milk (40%), refined sugar (8.5%) and filtered water *qsp*, as previously described (17) for a final mixture containing 65% total carbohydrates (25% sucrose), 12.3% proteins and 4.3% total lipids, totalling 3.48 kcal/g. The standard laboratory diet (Nuvital, Nuvilab, Brazil) contained 55.4% total carbohydrates (10% sucrose), 21% proteins and 5.2% total lipids, totalling 3.52 kcal/g.

### Study Design

Weaned female Wistar rats were provided by the Animal Facility House of UFMA and randomized into two groups: standard laboratory diet (CTR, n = 8) and HSD (n = 8) groups. Dietary intervention started at postnatal day (pnd) 21 and continued until euthanasia, at pnd 120. Animals were weekly monitored for: weight gain, food intake and feed efficiency. In order to verify the onset of puberty, the vaginal opening was observed daily, starting at pnd 21. From the pnd 90 until euthanasia, the phases of the estrous cycle were determined as described below. During the last week of dietary intervention, animals were submitted to glucose and insulin tolerance tests. The Lee Index ((body weight (g)1/3 ÷ naso-anal length (cm)) × 1000) was used to assess body mass index (18) at pnd 120. Between pnd 118 and 122, rats in the oestrous phase were fasted for 8-h and anesthetized (70 mg/kg ketamine and 10 mg/kg xylazine, i.p.) for blood and tissue collection. Blood samples were processed for serum separation, which were stored for later biochemical analyses. Morphometric analysis of organs such as liver, pancreas, ovaries and retroperitoneal and visceral fat depots were also performed. In addition, ovaries and attached fat pads (periovarian fat) were collected, fixed in 4% v/v paraformaldehyde and kept on 70% v/v ethanol for histological analysis as described previously (19).

### Assessment of Vaginal Opening and Estrous Cyclicity

In order to determine the beginning of sexual maturity, the vaginal opening was observed daily, beginning at pnd 21. The estrous cycle was evaluated daily starting at pnd 90 until pnd 120, between 4:00 pm and 6:00 pm, according to Marcondes et al. (20) with minor adaptations. Briefly, vaginal smears were obtained using a plastic pipette containing 10 μL of saline solution (0.9% NaCl v/v). The collected material was placed on fresh glass slides and observed with an optical microscope with 10x and 40x lenses. The proportion of nucleated epithelial cells, cornified cells or leukocytes was used to determine the different stages of the estrous cycle (proestrus, oestrus, metestrus and diestrus) as described by Hubscher et al. (21).

### Oral Glucose Tolerance (OGTT) and Insulin Tolerance (ITT) Tests

For the OGTT, animals were fasted for 8 hours and subsequently a glucose bolus (4 g/kg body weight) was administered by oral gavage. Blood was collected through puncture of the caudal vein and glycaemia assessed using a glucometer (Accu-Chek Active^®^, Roche, USA) at 0 (before glucose administration), 15, 30, 60 and 120 minutes after glucose administration. For ITT, fed animals received recombinant human insulin (2 IU/kg preheated to 36 °C). Glycaemia was measured at 0 (before insulin administration), 3, 5, 10, 15 and 20 minutes after insulin administration. The constant rate of glucose disappearance (kITT) was calculated from the regression slope obtained from the log-transformed glucose values between 3 and 20 minutes after insulin injection (22).

### Biochemical Analyses

Upon euthanasia, blood aliquots were collected and centrifuged (1372 × *g* for 10 min) to obtain serum. Fasting triglyceride and cholesterol levels were detected in serum using spectrophotometric test kits following the manufacturer’s guidelines (Labtest, Lagoa Nova, MG, Brasil). Fasting and fed blood glucose levels were defined as time 0 of GTT and ITT assays, respectively. The TyG index was used to infer insulin resistance using the formula: Ln [glucose (mg/dL) x triglycerides (mg/dL)/2] (23).

### Ovarian Histology

Rats at the oestrous phase were euthanized and the right ovary and surrounding fat pads were removed, cleaned, fixed in 4% paraformaldehyde for 24 hours and kept in 70% ethanol. The right ovary was immersed in paraffin, cut into 6 µm slices and stained with hematoxylin-eosin (HE). Since an oocyte has a diameter of 20 to 30 µm, we analysed one section every six cuts, therefore ensuring a 36 µm distance to prevent us from double-counting the same follicles. On average, nine slices in three sections were analysed per ovary. Full details on the classification of follicles, definition of atresia and follicular cysts can be found in previous reports of our lab (19, 24). Representative images of primordial, primary, secondary, antral and cystic follicles are presented in **Supplementary Figure 1**. A single-blinded competent researcher performed the histological analyses and the follicle count was divided by the number of sections analysed to obtain an average number of follicles per section.

### Periovarian Adipose Tissue Histology

The fat pads surrounding the right ovary were cut into 6 µm sections and stained with HE. Histology of adipocytes was performed as described previously (25). At least 100 adipocytes were analysed per animal. Representative photos were taken using an AxionVision (AxioVs40x64 V 4.9.1.0, Carl Zeiss GmBH Microscopy) and frequency distribution calculated according to adipocyte area.

### Ecologic Study

Data were collected from the Global Health Data Exchange, the Institute for Health Metrics and Evaluation, the Brazilian Institute of Geography and Statistics (IBGE) and the Brazilian Ministry of Health. Data were acquired for the 26 states and the Federal District from 2000 to 2017, totalling 459 state-year observations. The dependent variable was the prevalence of PCOS per 100,000 habitants, while the main independent variable was the summary exposure value (SEV) for diet high in sugar-sweetened beverages, as a surrogate for high sucrose intake. Control variables consisted of gross domestic product (GDP) in Brazilian Reais (R$) per capita, SEV for high LDL-cholesterol and SEV for high fasting glycaemia. Prevalence and SEV data were collected for women at reproductive age (15 to 49 years) and were defined and calculated by the global burden of diseases (GBD) study group (26). Detailed information with regards to data sources and definition of variables can be found in **Supplementary Table 1** and **Supplementary Table 2**. In addition, state- and year- fixed effects models were used to control for unobserved state-invariant and time-invariant heterogeneity, while increasing the robustness of the model.

Therefore, the fixed effects model used can be written as:

$$PCOS_{lt}=\alpha +\;\beta_{1}HighSugar_{lt}+\beta_{2}LDL_{lt}+\;\beta_{3}HG_{lt}+\;\beta_{4}GDP_{lt}+L_{t}+\;T_{l}+\;\epsilon_{lt}$$

Where PCOS*_lt_* is the prevalence of PCOS in state *l* year *t*, HighSugar*_lt_* is the SEV of diet high in sugar-sweetened beverages in state *l* year *t*, LDL*_lt_* is the SEV of high LDL-cholesterol in state *l* year *t*, HG*_lt_* is the SEV of hyperglycaemia in state *l* year *t*, GDP*_lt_* is the GDP per capita in state *l* year *t*, *L* is state fixed effect and *T* is time fixed effect. All variables were log-transformed to facilitate interpretation of estimates and ensure normal distribution.

A lag analysis was performed to test if the consumption of sugar-sweetened beverages in a given state was associated with the prevalence of PCOS in that same state 10 years later. This was performed using prevalence of PCOS in women aged 35-39 years and 40-44 years. The 10-year lag and age brackets were used due to restrictions on data collected from the GBD database.

The lagged fixed effects model can be written as:

$$PCOS_{lt}=\alpha +\;\beta_{1}HighSugar_{l-10t}+\beta_{2}LDL_{l-10t}+\;\beta_{3}HG_{l-10t}+\;\beta_{4}GDP_{lt}+L_{t}+\;T_{l}+\;\epsilon_{lt}$$

Where *l*–10*t* denotes the SEV of risk factors for state *t* in year *l* minus 10.

### Statistical Analysis

The results are expressed as mean ± SEM for rodent data and coefficient ± 95% confidence interval (CI) for the ecologic study. For the rodent study data were analysed using unpaired Student’s t test or two-way ANOVA, with Tukey’s post-test and n=6-8, except for ITT (n=5) and the histology of periovarian fat (n=4). The sample size for the animal study was calculated using the free software for sample calculation: G*Power 3.1 (Heinrich-Heine University Düsseldorf, Düsseldorf (NRW), Germany). The sample size of eight animals per group was found by inserting in the software sample power of 80% (1 − β = 0·8), significant level of 5% (α = 0·05), and parameters from previous studies on the effects of MSG on the number of ovarian cysts, antral follicles, and adipocytes area (19, 24). For the ecologic study, least squares multiple linear regression models were run and n=459 state-year observations. The differences were significant when p <0.05. The analyses were performed using the Graphpad Prism 7.0 and 9.0 software (GraphPad, San Diego, USA).

## Results

### Early Exposure of HSD to Female Rats Induced Fat Accumulation With No Increase in Body Weight

**Figure 1:**
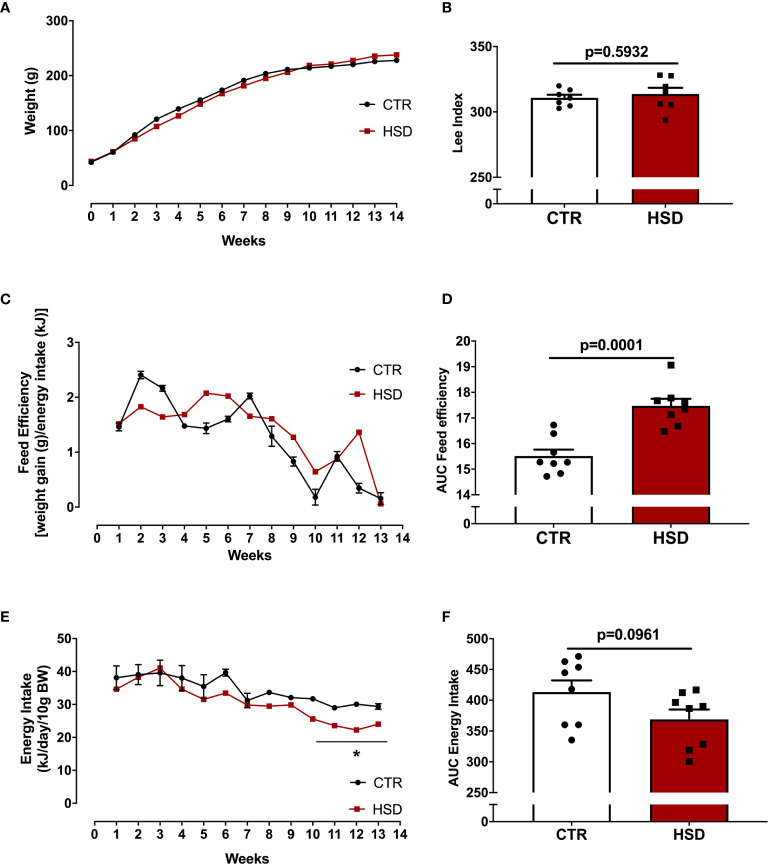
HSD ingestion results in no weight gain in spite of higher feed efficiency. **(A)** All animals were weighed weekly from pnd 21 (week 0) until pnd 120 (week 14). **(B)** Lee Index was calculated as described in Materials and Methods. **(C)** Feed efficiency was also monitored throughout dietary intervention. **(D)** Area under the curve (AUC) of feed efficiency. **(E)** Energy intake calculated as described in *Materials and Methods*. **(F)** AUC of energy intake. n=8 animals in each group. Graphs express mean ± SEM and bar graphs show individual values. *p < 0.05 *vs* CTR, assessed by two-way ANOVA with Tukey’s post-test. P-value presented for bar graphs and calculated through unpaired Student t-test.

To assess the impacts of HSD on the development of obesity, animals received HSD or CTR diet for a period of 14 weeks, from pnd 21 to 120. Body weight was assessed twice a week and fat pads were isolated at the end of the dietary intervention. As shown in **Figure 1**, HSD did not increase body weight or Lee’s Index over time (**Figures 1A, B**), despite a 17% increase in feed efficiency when compared to CTR (**Figures 1C, D**). There was no difference in energy intake (**Figures 1E, F**). Although there were no changes in body weight, HSD animals showed heavier retroperitoneal, visceral and periovarian fat depots, as shown in **Table 1**. Interestingly, HSD rats displayed lighter kidneys than did CTR rats, which should be addressed in the future. Together, these results suggest that HSD ingestion since weaning led to increased fat accumulation, in spite of no detectable changes in body weight and body mass index.

**Table 1:** Morphometric and hepatic lipid profile of CTR and HSD rats.

	CTR (mean ± SEM)	HSD (mean ± SEM)
**Morphometric features**		
Ovary (g/100 g)	0.098 ± 0.006	0.097 ± 0.008
Uterus (g/100 g)	0.229 ± 0.020	0.195 ± 0.019
Liver (g/100 g)	3.109 ± 0.074	3.064 ± 0.166
Visceral fat pads (g/100 g)	2.669 ± 0.265	4.088 ± 0.413*
Retroperitoneal fat pads (g/100 g)	1.30 ± 0.077	1.672 ± 0.110*
Periovarian fat pads (g/100 g)	353.30 ± 56.650	565.20 ± 46.320*
Heart (g/100 g)	0.296 ± 0.006	0.299 ± 0.005
Kidneys (g/100 g)	0.840 ± 0.032	0.719 ± 0.034*
Pancreas (g/100 g)	0.322 ± 0.025	0.340 ± 0.031
**Hepatic lipid profile**		
Triglycerides (mg/g Liver)	54.380 ± 2.437	54.940 ± 2.798
Total Cholesterol (mg/g Liver)	77.210 ± 4.478	82.280 ± 2.848

Results expressed as mean ± S.E.M. *p < 0.05 vs CTR compared with unpaired Student t-test. n=8 for CTR and HSD.

### HSD Alters Glucose Metabolism and Causes Insulin Resistance in Female Rats

**Figure 2:**
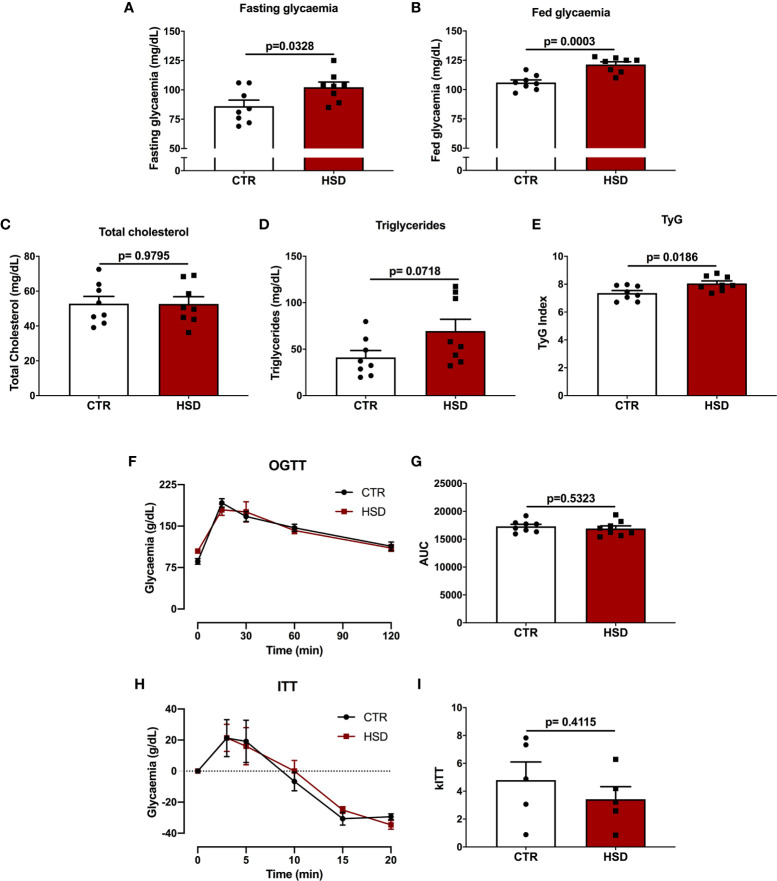
High-sucrose diet (HSD) alters glucose metabolism and causes insulin resistance in female rats. **(A)** Fasting glycaemia was calculated as time 0 of OGTT. **(B)** Fed glycaemia was calculated as time 0 of ITT. **(C)** Total cholesterol and **(D)** triglycerides were measured in fasting rats upon euthanasia (pnd 120). **(E)** TyG Index was calculated as described in Materials and Methods. **(F)** Glucose tolerance test (OGTT) was assessed in 8-hour fasted rats at times 0, 15, 30, 60 and 120 min. **(G)** Area under the curve (AUC) of OGTT. **(H)** Insulin tolerance test (ITT) assessed in fed animals at times 0, 3, 5, 10, 15 and 20 min. **(I)** kITT calculated as described in Materials and Methods. n=5-8 animals in each group. Graphs express mean ± SEM and bar graphs show individual values. P-values are shown in bar graphs and were calculated using unpaired Student t-test.

In order to determine the effects of HSD on the glucose-insulin axis of female rats, blood glucose (fasting and fed), cholesterol and triglycerides were measured. In addition, OGTT and ITT were performed to assess glucose tolerance and insulin sensitivity, respectively. As shown in **Figure 2**, the HSD group presented increased levels of both fasting (CTR 86.13 ± 5.16 *vs* HSD 102.30 ± 4.43, *p* = 0.03) and fed (CTR 106.0 ± 2.26 *vs* HSD 121.40 ± 2.30, *p* = 0.0003) blood glucose when compared to CTR rats (**Figures 2A, B**), however, there were no differences in cholesterol or triglyceride levels across groups (**Figures 2C, D**). HSD rats displayed increased TyG Index (CTR 7.36 ± 0.18 *vs* HSD 8.05 ± 0.18, *p* = 0.01), suggesting these animals presented IR (**Figure 2E**) in spite of similar tolerance to both glucose (**Figures 2F, G**) and insulin (**Figures 2H, I**) between groups. Therefore, the ingestion of HSD by female rats caused a mild metabolic dysfunction, characterized by increased fat accumulation, hyperglycaemia and IR. This could potentially damage the reproductive function of these animals.

### HSD Exposure Anticipates Puberty and Impairs Ovarian Follicular Development in Female Rats

**Figure 3:**
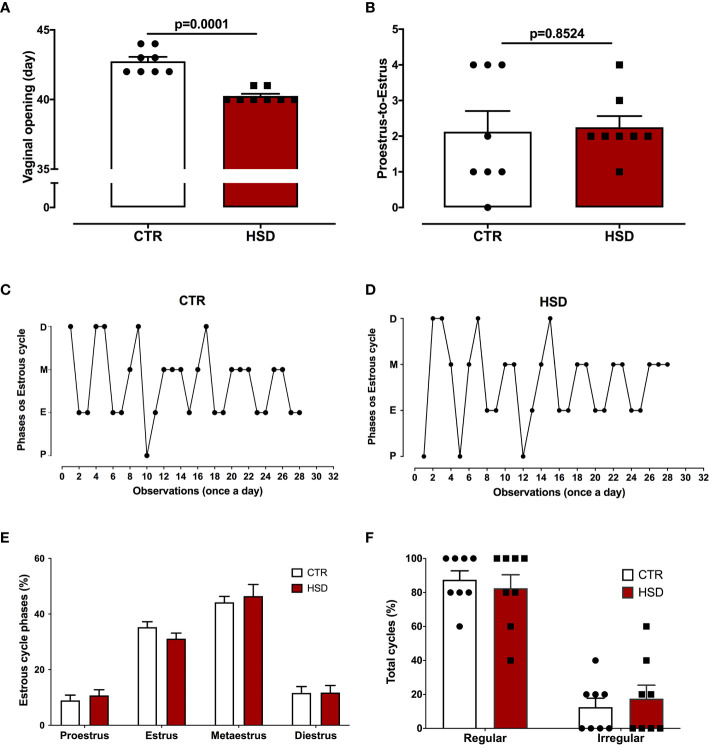
High-sucrose diet (HSD) exposure anticipates puberty. **(A)** Vaginal opening assessed daily, beginning at pnd 21. **(B)** Proestrus-to-estrus change as a surrogate for ovulation. **(C)** Oestrous cycles from a representative CTR rat, where proestrus (P), oestrus **(E)**, metestrus (M) and diestrus **(D)**. **(D)** Oestrous cycle from a representative HSD rat. **(E)** Percentage of each oestrous phase. **(F)** Percentage of regular or irregular cycles, as defined in Materials and Methods. n=8 animals in each group. Graphs express mean ± SEM and bar graphs show individual values. P-values are shown in bar graphs when significant (p < 0.05) and were calculated using unpaired Student t-test in **(A)** and two-way ANOVA in **(D, E)**.

Taking into account the influence of metabolic changes on the reproductive system, vaginal opening (indicative of the beginning of sexual maturity) and oestrous cycle were monitored (**Figure 3**). As shown in **Figure 3A**, the HSD group entered puberty 2.5 days earlier than CTR animals (CTR 42.75 ± 0.31 *vs* HSD 40.25 ± 0.16, *p <*0.0001), suggesting that HSD ingestion led to early puberty. In spite of earlier vaginal opening, HSD rats ovulated and cycled normally, similar to CTR animals (**Figures 3B–F**).

**Figure 4:**
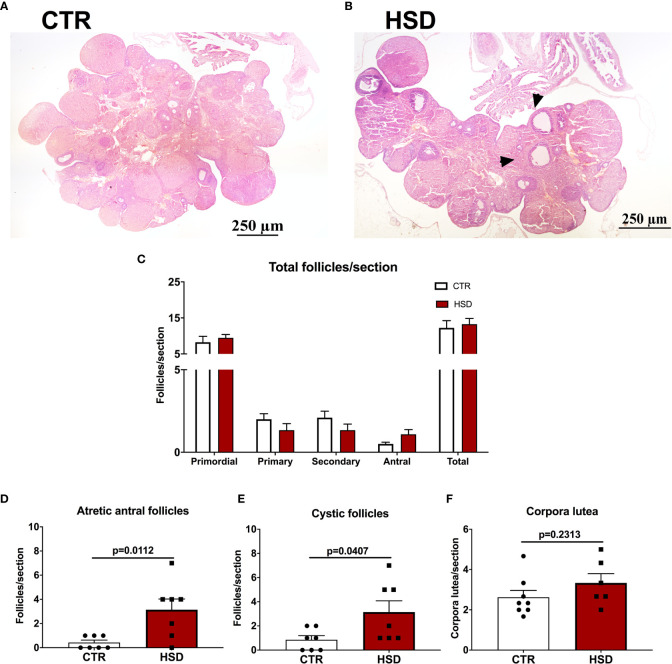
High-sucrose diet (HSD) ingestion causes ovarian dysfunction in female rats. Ovaries were analysed and follicles classified according to *Materials and Methods*. **(A)** Representative micrograph of an ovary from a CTR rat. **(B)** Representative micrograph of an ovary from an HSD rat, depicting cystic follicles (arrowheads). **(C)** Number of primordial, primary, secondary, antral and sum of all follicles (total) per section. **(D)** Number of atretic antral follicles per section. **(E)** Number of cystic follicles per section. n=7-8 animals in each group. **(F)** Number of corpora lutea per section. Graphs express mean ± SEM and bar graphs show individual values. P-values are shown in bar graphs when significant (p < 0.05) and were calculated using two-way ANOVA in **(C)** and unpaired Student t-test in **(D–F)**.

The ovarian histology was analysed to assess if the mild metabolic dysfunction observed in HSD rats were correlated with impairments in the development of ovarian follicles (**Figure 4**). Representative micrographs are presented in **Figures 4A, B**. There were no differences on the number of ovarian follicles between groups (**Figure 4C**). However, the HSD group showed a greater number of atretic antral follicles (**Figure 4D**) (CTR 0.19 ± 0.06 *vs* HSD 1.22 ± 0.28, *p* = 0.002) and cystic follicles (**Figure 4E**) (CTR 0.28 ± 0.11 *vs* HSD 1.04 ± 0.31, *p* = 0.04) when compared to ovaries from CTR rats. The number of corpora lutea was also unaltered in HSD rats when compared to those of CTR animals (**Figure 4F**). In spite of unaltered ovulation, these data suggest ovarian dysfunction similar to a PCOS-like phenotype in HSD rats, considering the 1) earlier vaginal opening, 2) increased atretic antral follicles and 3) increased number of ovarian cysts. These ovarian dysfunctions were associated with increased fat deposition and impaired glucose-insulin axis.

### PCOS-Like Features of HSD Rats Are Associated With Hypertrophy of Periovarian Adipocytes

**Figure 5:**
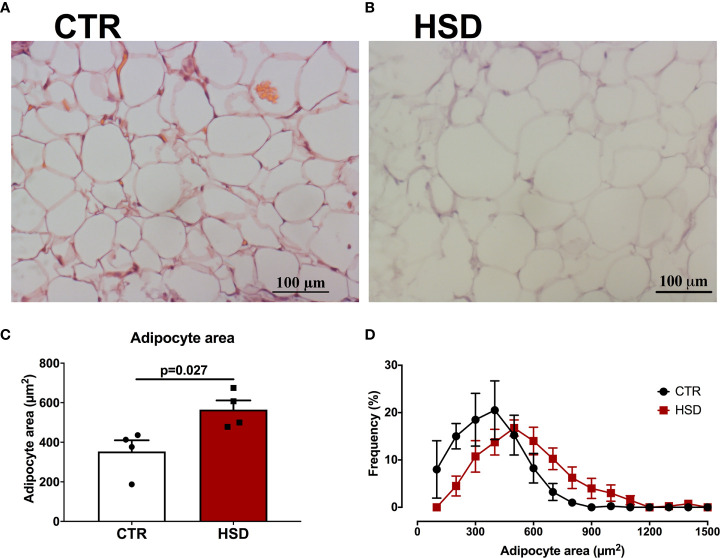
HSD rats present hypertrophy of periovarian adipocytes. Periovarian fat pads were analysed as described in Materials and Methods. **(A)** Representative micrograph of periovarian fat from a CTR rat. **(B)** Representative micrograph of periovarian fat from an HSD rat. **(C)** Mean adipocyte area of periovarian fat. **(D)** Frequency distribution per adipocyte area. n=4 animals in each group. Graphs express mean ± SEM and bar graphs show individual values. P-values are shown in bar graphs when significant (p < 0.05) and were calculated using Student t-test in **(C)**.

Previous studies of our group have shown that the periovarian fat pad is correlated with PCOS-like ovarian dysfunctions (19, 24), such as the ones presented by HSD rats. Therefore, we sought to analyse if a similar phenotype was present in HSD animals (**Figure 5**). According to the histological analysis of periovarian fat, it was observed that HSD contributed to a larger average area of adipocytes, increasing 59% when compared to CTR (CTR 353.30 ± 56.65 *vs* HSD 565.20 ± 46.32 μm^2^, *p* = 0.275) (**Figure 5C**). This is reinforced by the frequency distribution graph showing the periovarian fat of HSD animals presented a higher frequency of larger adipocytes (**Figure 5D**). Collectively, these data indicate that the mild metabolic dysfunction caused by HSD was associated with PCOS-like features, which were also related to the hypertrophy of periovarian fat.

### Consumption of Sugar-Sweetened Beverages Is Correlated With Prevalence of PCOS

**Figure 6:**
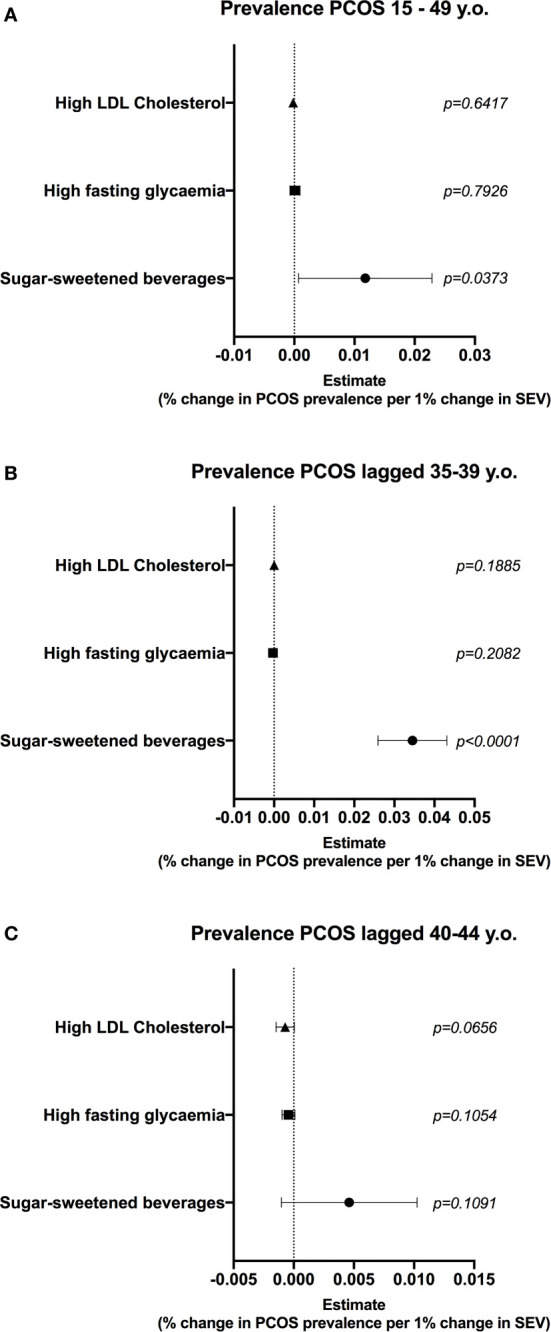
Consumption of diet high in sugar-sweetened beverages is correlated with prevalence of PCOS in women at reproductive age. A multiple linear regression model (459 state-year observations) was used to test the correlation between diet high in sugar-sweetened beverages and prevalence of polycystic ovary syndrome (PCOS). State and year fixed effects were included in the model. Gross domestic product (GDP) per capita was used to control for income. **(A)** Correlation between summary exposure value (SEV) of risk factors and prevalence of PCOS in women at reproductive age. **(B)** Correlation between SEV of risk factors and prevalence of PCOS 10 years later, in women aged 35-39. **(C)** Correlation between SEV of risk factors and prevalence of PCOS 10 years later, in women aged 40-45. Graphs express mean ± 95% confidence interval and *p* is shown for each SEV.

To further explore the interaction between consumption of HSD and ovarian function, we performed a fixed-effect regression model using data from Brazilian states and tested the correlation between diet high in sugar-sweetened beverages (surrogate for HSD intake) and the prevalence of PCOS (surrogate for ovarian dysfunction) in women at reproductive age. Controls for income and other risk factors, namely hyperglycaemia and dyslipidaemia were used, since similar alterations were seen in HSD rats. The prevalence of PCOS in women at reproductive age was positively correlated with the consumption of sugar-sweetened beverages (**Figure 6A**) (0.011, 0.006 – 0.022 95%CI), while there were no associations for other risk factors. Interestingly, there was a positive correlation between the consumption of sugar-sweetened beverages in women aged 25-29 and the prevalence of PCOS in women after a 10-year lag period, aged 35-39 (**Figure 6B**) (0.03, 0.02 – 0.04). The magnitude of this association was three times higher than that of the non-lagged analysis. Moreover, the lag effect was lost when an older population was used (**Figure 6C**). There were no associations for other risk factors and the prevalence of PCOS in the lagged analyses. Altogether, these data suggest a positive association between the consumption of sugar-sweetened beverages and deteriorated ovarian function in the Brazilian population. Although unable to infer causality, this reinforces the need to explore the effects of HSD consumption in women.

## Discussion

Here we hypothesized that consumption of HSD, at a proportion similar to that consumed in Latin American countries (7), would lead to ovarian dysfunction in rats. This issue was addressed in two ways: first by giving HSD to female rats and measuring the ovarian repercussions of such intervention, and second by analysing the correlation between consumption of sugar-sweetened beverages and prevalence of PCOS in a Latin American country, i.e. Brazil. We show that HSD led to mild metabolic dysfunctions in female rats, while there were earlier vaginal opening, increased presence of ovarian cysts and hypertrophy of periovarian adipocytes, suggesting PCOS-like features. However, we could not ascertain all features of this syndrome in our experimental model. In the ecologic study, there was a positive correlation between the prevalence of PCOS (surrogate for ovarian dysfunction) and consumption of diet high in sugar-sweetened beverages (a surrogate for HSD intake). Altogether, the consumption of HSD resulted in deleterious impacts to the ovarian milieu, which could be associated with an increased prevalence of ovarian dysfunction in the population.

Despite not influencing body weight gain, HSD rats displayed greater food efficiency compared to CTR. These findings are in agreement with previous studies that demonstrated the association between HSD and rises in circulating leptin levels, a hormone responsible for satiety and, consequently, decreased food intake (27). Moreover, fasting and postprandial hyperglycemia, coupled with IR found in the HSD group, may have contributed to the reduction of energy consumption, given that higher glucose levels lead to the production of malonyl-CoA. This peptide, in turn, negatively modulates the energy balance through the anorexigenic orexigenic neuropeptide system (28), a mechanism activated by high-sucrose but not high-fat diets (29). Notwithstanding, adipose tissue accumulation in the HSD group was increased in all the assessed fat pads, which could be a consequence of excess circulating levels of sucrose-derived fructose. Indeed, fructose is massively up-taken by adipocytes, where it is metabolised into triglycerides and contributes to adipocyte hypertrophy (30). Collectively, this set of data demonstrates that post-weaning exposure of female rats to HSD leads to consistent metabolic disturbances, although at a lesser extent than those observed in age-matched male rats (31).

In addition to metabolic changes, our study showed reproductive disorders in HSD animals. The first striking observation was that HSD rats entered puberty earlier than CTR, despite being exposed to the diet for a short period of time, which could be due to increased periovarian adipose tissue accumulation. In immature rats, the periovarian fat has been shown to synthesize oestrogen at a greater capacity than the ovary, serving as a primary source of this hormone in the prepubertal period, thus leading to the onset of puberty (32). Notwithstanding, Connor et al. (33) suggested that the mechanism by which fat accumulation influences early puberty is mainly due to excessive energy supply. Although we have not assessed periovarian fat pad on HSD rats at the time of puberty onset, a previous report from our lab showed that short-term exposure to HSD significantly augmented the periepididymal fat accumulation in age-matched male mice (34). In women, it has been shown that precocious puberty is associated with MetS risk factors, such as IR and excessive body fat (35). Moreover, the consumption of sugary drinks has been associated with early menarche (36). These data collectively show that early introduction of HSD hastens feminine sexual maturation.

In line with early puberty onset, HSD rats displayed increased cystic and atretic antral ovarian follicles. Likewise, Nino et al. (37) found that a high-carbohydrate diet was able to increase the number of atretic follicles in rats. Furthermore, Roberts et al. (38) have shown that animals fed a high-fat, high-sugar diet had higher frequency of cystic follicles when compared to control animals. It is yet unclear how the ingestion of HSD or other types of hypercaloric diets lead to ovarian dysfunction, however feasible underlying mechanisms include: hyperinsulinemia culminating in higher production of ovarian androgens in synergy with the luteinizing hormone (10), increased expression of anti-mullerian hormone in granulosa cells (19) and increased production of reactive oxygen species (39). The identification of underlying mechanisms linking increased dietary intake of sugars and ovarian dysfunction will lead to novel targets to treat these conditions.

Clinically, the main endocrinopathy linked to impaired follicular health is PCOS, a condition that affects 5% to 15% of women at reproductive age (40). Previous studies conducted by our lab have shown that exposure to monosodium L-glutamate (MSG) was able to induce a PCOS-like phenotype (19). This was thought to be due to hyperinsulinemia found in MSG rats, since ovaries remain sensitive to insulin (41). The ovarian dysfunction phenotype observed in HSD rats, as well as in other models using high-carbohydrate diets may help identify risk factors and underlying mechanisms for PCOS in women.

To translate findings in rodents to a broader context, we have tested if there was a correlation between the consumption of sugar-sweetened beverages (as a proxy for HSD) and the prevalence of PCOS (proxy for ovarian dysfunction) in the Brazilian population. It was evident that there was a positive correlation between consumption of sugar-sweetened beverages and prevalence of PCOS. Interestingly, there was a lag effect between the consumption of sugar-sweetened beverages at 25-29 years and the prevalence of PCOS at 35-39 years that was lost when an older bracket of the population was analysed. This is in line with the programming effect of HSD consumption at younger ages, such as demonstrated for HSD rats. In parallel, it has been reported that adolescents with PCOS tend to consume a large amount of foods rich in sugar (42), while, in developing countries, such as Brazil, over 60% of the population was reported to consume excessive amounts of added sugars (7). Indeed, a relationship was identified between women with PCOS and the consumption of foods with a high glycemic index (43). These results reinforce the positive correlation between the consumption of sugary drinks and the prevalence of PCOS found in our study.

Some limitations of the current study need to be addressed in the future. First, while we identified early puberty in HSD rats, we were unable to explore mechanisms that led to this event. A feasible hypothesis is that HSD could cause the hypertrophy of periovarian fat in prepubertal rats, as discussed above. Future studies should assess if periovarian fat pads are altered in prepubertal rodents exposed to HSD. Second, further studies are needed to extend our observations *in vivo* using a larger sample size. Third, we were unable to ascertain if HSD rats developed PCOS due to lack of hormonal levels and we suggest this to be explored in the future. Finally, the ecologic study does not allow causal inference between the consumption of sugary drinks and the prevalence of PCOS. While other factors could influence the prevalence of PCOS, controls for income and fixed effects models were employed to account for some externalities that could influence the prevalence of PCOS. Nonetheless, it is imperative to address in the future if HSD can lead to PCOS in women.

In conclusion, data herein presented show that early exposure to HSD causes metabolic and ovarian dysfunctions in rats, which could have implications to the prevalence of PCOS in women. More studies are needed to address the link between HSD consumption and PCOS development. Moreover, our study suggests that early introduction of added sugars in the diet, particularly following breastfeeding cessation, is detrimental to female reproductive health. Therefore, sucrose-limiting policies could potentially hinder the deleterious impacts described above.

## Data Availability Statement

The raw data supporting the conclusions of this article will be made available by the authors, without undue reservation.

## Ethics Statement

Ethical review and approval were not required for the study on human participants, because data were collected from a public database, in accordance with the local legislation and institutional requirements. Written informed consent for participation was not required for this study in accordance with the national legislation and the institutional requirements. The animal study was reviewed and approved by the Ethical Committee on Animal Use and Welfare at UFMA, ruling number 23115.007440/2016-71.

## Author Contributions

GM, TC, RB, and CV have performed experiments and analysed data. JS has analyzed data and written the manuscript. AP has supervised experiments, conceived the study, analyzed data, and reviewed the manuscript. RG has performed experiments, conceived the study, analyzed data, and written the manuscript. All authors contributed to the article and approved the submitted version.

## Funding

This work received financial support from the University of Reading, Fundação de Amparo à Pesquisa e ao Desenvolvimento Científico, Tecnológico e Inovação do Estado do Maranhão – FAPEMA (IECT 05593/18) and Coordenação de Aperfeiçoamento de Pessoal de Nível Superior – Brasil (CAPES) – Finance Code 001. GM received a PIBIC fellowship from FAPEMA. JS, TC, RB, and CV received a Masters fellowship from CAPES. AP received a Researcher fellowship from CNPq (308163/2019-2).

## Conflict of Interest

The authors declare that the research was conducted in the absence of any commercial or financial relationships that could be construed as a potential conflict of interest.
