# Supplementary material for: Early Exposure to High-Sucrose Diet Leads to Deteriorated Ovarian Health
Source: Front Endocrinol (Lausanne). 2021 Apr 19;12:656831. doi: 10.3389/fendo.2021.656831 (PMC8092397; doi:10.3389/fendo.2021.656831)
Supplement: Supplementary file 1 [file DataSheet_1.pdf]

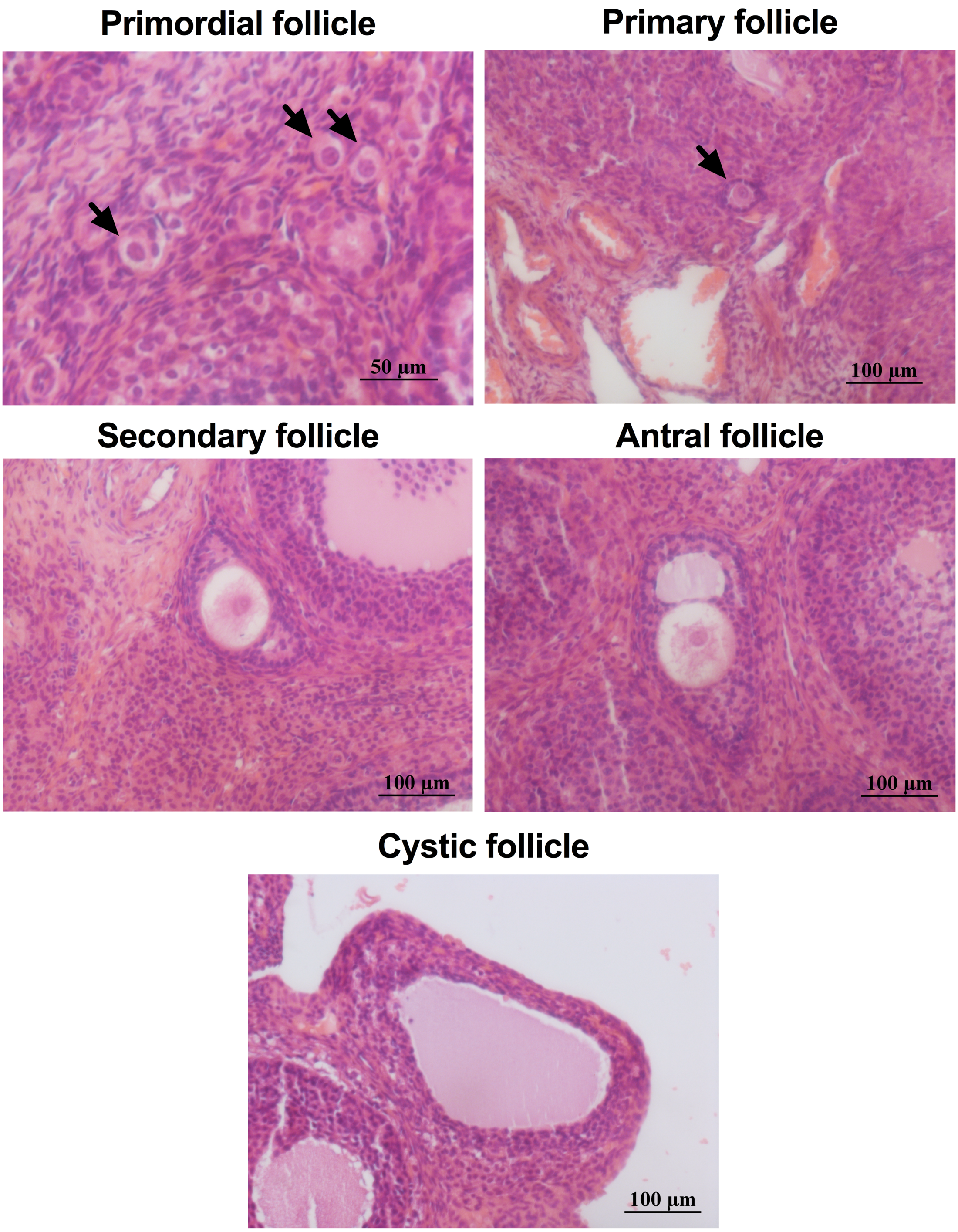


**Supplementary Figure 1. Representative images of primordial, primary, secondary, antral and cystic follicles.** Representative microphotographs of the ovaries of our rats were obtained using a camera attached to an optical microscope and ovaries were stained with HE.

Supplementary Table 1: Descriptive information on variables and data sources explored across 26 Brazilian states from 2000 to 2017.

| Variable name | Unit | Source |
|---|---|---|
| **Socioeconomic indicators** | | |
| GDP per capita | Reais (R$) | Brazilian Institute of Geography and Statistics (IBGE) |
| **Health outcomes and risks** | | |
| Prevalence of polycystic ovary syndrome | Cases per 100,000 habitants | Global Health Data Exchange/GBD Results Tool |
| SEV of diet high in sugar-sweetened beverages | Risk-adjusted prevalence, from 0 to 1 | Global Health Data Exchange/GBD Results Tool |
| SEV of high fasting glycaemia | Risk-adjusted prevalence, from 0 to 1 | Global Health Data Exchange/GBD Results Tool |
| SEV of high LDL-cholesterol | Risk-adjusted prevalence, from 0 to 1 | Global Health Data Exchange/GBD Results Tool |

GDP: Gross domestic product. SEV: Summary of exposure value. GBD: Global Burden of Disease study.

Supplementary Table 2: Glossary of metrics and definition of variables used in the ecological study.

| Metric and variables | Definition |
|---|---|
| GDP per capita | It is the gross domestic product divided by the midyear population. GDP is the sum of the gross value added by all resident producers in the economy plus any product taxes and minus any subsidies not included in the value of the products. It is calculated without making deductions for depreciation of fabricated assets or for depletion and degradation of natural resources. |
| Prevalence | The total number of cases of a given disease in a specified population at a designated time. It is differentiated from INCIDENCE, which refers to the number of new cases in the population at a given time. |
| Summary Exposure value (SEV) | A measure of a population's exposure to a risk factor that takes into account the extent of exposure by risk level and the severity of that risk's contribution to disease burden. SEV takes the value zero when no excess risk for a population exists and the value one when the total population is at the highest level of risk; we report SEV on a scale from 0% to 100% to emphasize that it is risk-weighted prevalence. |
| Polycystic Ovary Syndrome | PCOS is defined according to NIH criteria (as recommended by American College of Obstetricians and Gynecologists [ACOG]), which includes chronic anovulation and hyperandrogenism (established by hormone measurements or clinical findings) in women in whom secondary causes have been excluded. PCOS included the following ICD-10 code: E28.2. |
| Diet high in sugar-sweetened beverages | Diet high in sugar-sweetened beverages is defined as any intake (in grams per day) of beverages with ≥50 kcal per 226·8 gram serving, including carbonated beverages, sodas, energy drinks, and fruit drinks, but excluding 100% fruit and vegetable juices. |
| High fasting plasma glucose | High fasting plasma glucose is defined as serum fasting plasma glucose of greater than 4·8–5·4 mmol/L. |
| High LDL-cholesterol | We estimated blood concentration of low-density lipoprotein (LDL) in units of mmol/L. We used a TMREL with a uniform distribution between 0·7 and 1·3 mmol/L. |

ICD: International classification of diseases. TMREL: Theoretical minimum risk exposure level.
